# Understanding the complex-I-ty of metformin action: limiting mitochondrial respiration to improve cancer therapy

**DOI:** 10.1186/s12915-014-0082-4

**Published:** 2014-10-24

**Authors:** Alba Luengo, Lucas B Sullivan, Matthew G Vander Heiden

**Affiliations:** The Koch Institute for Integrative Cancer Research and Department of Biology, Massachusetts Institute of Technology, Cambridge, MA 02139 USA; Dana-Farber Cancer Institute, Boston, MA 02115 USA

## Abstract

Metformin has been a first-line treatment for type II diabetes mellitus for decades and is the most widely prescribed antidiabetic drug. Retrospective studies have found that metformin treatment is associated with both reduced cancer diagnoses and cancer-related deaths. Despite the prevalence of metformin use in the clinic, its molecular mechanism of action remains controversial. In a recent issue of *Cancer & Metabolism*, Andrzejewski *et al.* present evidence that metformin acts directly on mitochondria to inhibit complex I and limits the ability of cancer cells to cope with energetic stress. Here, we discuss evidence that supports the role of metformin as a cancer therapeutic.

See research article: http://www.cancerandmetabolism.com/content/2/1/12.

The biguanide metformin is an antihyperglycemic agent used to treat type II diabetes. Metformin decreases blood glucose levels by suppressing liver gluconeogenesis and stimulating glucose uptake in skeletal muscle and adipose tissues. Metformin is prescribed to over 120 million people, providing a wealth of epidemiological data. Retrospective studies have found that metformin treatment is associated with diminished tumorigenesis, with a recent meta-analysis of these studies reporting a 31% reduction in cancer incidence and a 34% reduction in cancer-specific mortality across many tumor types [1]. While these findings are provocative, it remains controversial whether the effects of metformin on improving cancer outcomes are a result of altered whole body metabolism or if metformin can act in a cell autonomous manner. Indeed, the anticancer action of metformin can be divided into two categories: ‘indirect effects’ resulting from systemic changes in metabolism, such as reduced concentrations of blood glucose and insulin, and ‘direct effects’ on tumor cells (Figure 1). Importantly, both could act synergistically or have differential importance depending on the cancer context. The molecular mechanisms by which metformin can impact tumor biology are an area of active research and clinical trials are ongoing to define the role of metformin in cancer treatment.

**Figure 1:**
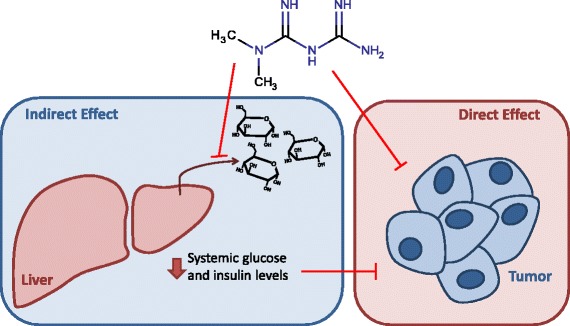
**Indirect and direct effects of metformin on tumors.** Metformin can suppress tumor progression by modulating metabolic whole body physiology or by acting directly on cancer cells. Metformin diminishes hepatic glucose output leading to lower systemic glucose and insulin levels, which could impair malignant growth indirectly without requiring accumulation of metformin in the tumor. Alternatively, metformin can act on cancer cells directly, inhibiting cancer progression by suppressing mTOR signaling, mitochondrial glucose oxidation, and/or reducing stability of HIF under hypoxic conditions.

## Metformin action on cells and tissues

**Figure 2:**
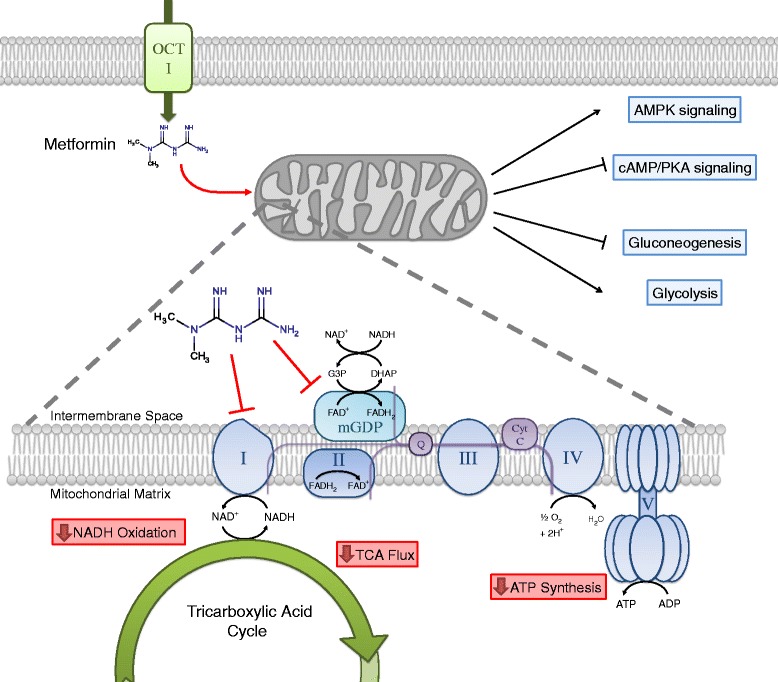
**Cellular consequences of metformin action at the mitochondria.** Metformin enters the cell by organic cation transporter 1 (OCT1), where it then accumulates in the mitochondria. There, metformin inhibits complex I of the electron transport chain and mGDP, resulting in decreased NADH oxidation. Decreased electron chain activity suppresses tricarboxylic acid (TCA) cycle flux and decreases mitochondrial ATP synthesis. These actions result in increased AMPK signaling, decreased cAMP/PKA signaling, decreased gluconeogenesis and increased glycolysis.

Although the molecular underpinnings of metformin action remain an area of active investigation, the best described mechanism is inhibition of complex I, the first component of the mitochondrial electron transport chain (Figure 2). Complex I inhibition by metformin interrupts mitochondrial respiration and decreases proton-driven synthesis of ATP, causing cellular energetic stress and elevation of the AMP:ATP ratio. These changes result in allosteric activation of 5’-AMP-activated protein kinase (AMPK), a primary metabolic sensor. Hepatic AMPK activation can inhibit gluconeogenesis and activates glycolysis [2]. AMPK activation in the muscle can also increase glucose consumption, and is another potential site of metformin action [3]. Both of these consequences of metformin can lower blood glucose and contribute to therapeutic benefit in in type II diabetes.

Though AMPK was once considered the primary executor of metformin antidiabetic action, genetic loss-of-function studies in mice have indicated that hepatic expression of AMPK and its upstream activating kinase LKB1 may not be absolutely required for suppression of gluconeogenesis by metformin [4]. An AMPK-independent mechanism has been proposed, by which metformin antagonizes glucagon-dependent cyclic AMP (cAMP) signaling [5]. Glucagon activates adenylyl cyclase to produce cAMP and stimulate cAMP-dependent protein kinase (PKA) signaling. PKA activation decreases fructose-2,6,-bisphosphate levels, thereby favoring gluconeogenesis in the liver and increasing blood glucose levels. Metformin opposes glucagon action because inhibition of the mitochondrial electron transport chain elevates cytosolic AMP:ATP ratios, which in turn abrogates cAMP production [5]. Therefore, inhibition of PKA signaling by metformin may be another mechanism for inhibition of hepatic gluconeogenesis and reduced hyperglycemia in patients treated with the drug.

In addition to targeting complex I, metformin has recently been shown to inhibit mitochondrial glycerol-phosphate dehydrogenase (mGPD; Figure 2) [6]. mGPD transports cytosolic reducing equivalents from NADH into the mitochondria via the glycerol-phosphate shuttle. Interestingly, inhibition of mGPD and complex I both compromise the ability of mitochondria to oxidize cytosolic NADH and decrease the entry of these reducing equivalents into the electron transport chain. Therefore, both complex I and mGPD inhibition are expected to have similar downstream consequences on cellular bioenergetics.

Regardless of how metformin disrupts mitochondrial respiration, the resulting energy stress, changes in cofactor balance, and reliance on alternative metabolic pathways to obtain ATP could have several effects that contribute to the therapeutic benefits of this drug. AMPK activation, PKA inhibition, and redox stress may act separately or in concert to repress hepatic gluconeogenesis and stimulate insulin sensitivity. There are many ways in which metformin might act across tissues to alter metabolic physiology, and these complexities make it challenging to understand how metformin improves outcomes in cancer patients.

## Role of metformin in cancer therapy

The association between metformin and decreased cancer risk may be a result of the antidiabetic effects of the drug. Increased glucose consumption is a hallmark of many cancer cells, and increased blood glucose and insulin levels seen in type II diabetes are associated with worse cancer prognosis [7]. Metformin improves glycemic control and lowers insulin levels, which can influence other hormones and cytokines, including insulin-like growth factor 1 (IGF-1), a known mitogen that has been shown to promote carcinogenesis. Most of the patients in existing retrospective metformin studies were diabetic, so ongoing efforts to define the benefit of metformin in non-diabetic cancer patients will provide important insight into the role indirect metformin effects play in improved cancer outcomes.

Metformin affects systemic metabolism, but can also influence cell autonomous energetics. Metformin treatment leads to energy stress and AMPK activation in cancer cells, which can impair proliferation by suppressing anabolic processes and growth signaling through mammalian target of rapamycin (mTOR). Increased mTOR activation has been implicated in a wide variety of different cancers, and AMPK-mediated attenuation of anabolic metabolism and/or mTOR signaling by metformin may contribute to the antitumor effects of metformin in some situations. However, LKB1-deficient tumors, which are compromised in their ability to activate AMPK, are particularly sensitive to biguanides [8]. This suggests that induction of energy stress may account for some of the ability of metformin to limit cancer progression.

The findings of Andrzejewski *et al.* [9] support the observation that metformin-mediated energy imbalances can cause toxicity in cells. They demonstrate that metformin acts directly on mitochondrial complex I in cancer cells, limiting respiration and blocking glucose entry into the tricarboxylic acid cycle. It has long been known that malignant cells avidly consume glucose and preferentially metabolize glucose to lactate. However, studies of tumor metabolism *in vivo* have suggested that some tumor cells also oxidize glucose in the mitochondria [10]. Since metformin prevents mitochondrial glucose metabolism, metformin might be particularly effective in tumors that rely on glucose oxidation via the tricarboxylic acid cycle.

Decreased vascularization of tumors affects the availability of both glucose and oxygen and may influence how glucose is used by cancer cells *in vivo*. Stabilization of hypoxia-inducible factors (HIFs) is a critical part of the metabolic adaptation to low-oxygen and activation of HIFs is reported in many cancers [11]. Metformin has recently been shown to reduce hypoxia-induced HIF1α stabilization and diminish expression of HIF target genes in tumors [12], suggesting that metformin may serve a therapeutic role in cancers that are dependent on HIF signaling for survival in hypoxic environments. A better understanding of the signaling and metabolic effects of metformin on cancer cells will help define other tumor contexts likely to be sensitive to metformin.

Despite increasing evidence that metformin can have profound effects on cancer cells, it is not well understood if the drug accumulates to sufficient levels to cause these direct effects in patient tumors. The liver is a clear site of drug action, as it is exposed to high levels of oral metformin and expresses the metformin transporter OCT1, facilitating uptake and allowing for drug accumulation in this tissue. At metformin doses prescribed to diabetic patients, the circulating levels in plasma and peripheral tissues are orders of magnitude below the concentrations used *in vitro* to elicit a biological effect in cancer cells. How the concentrations used *in vitro* translate to the effects on tumor tissues remains controversial, but some studies have shown that metformin induces cancer autonomous metabolic changes *in vivo*. Metformin has been shown to activate AMPK in intestinal tumors, even in the absence of changes in organismal blood glucose or insulin levels [13]. In another study, tumors engineered to express a surrogate for complex I that is refractory to metformin are resistant to the drug *in vivo* [12]. These data argue that the direct effect of metformin on mitochondrial electron transport can be relevant for tumor growth in a cell autonomous manner. A better understanding of the pharmacokinetic properties and the molecular mechanisms of metformin will better define how best to use this antidiabetic drug in cancer therapy.
